# Effect of implementation intention on walking in people with diabetes: an experimental approach

**DOI:** 10.11606/s1518-8787.2020054002024

**Published:** 2020-10-23

**Authors:** Marco Antonio Vieira da Silva, Thaís Moreira São-João, Marilia Estevam Cornelio, Fábio Luiz Mialhe

**Affiliations:** I Universidade Estadual de Campinas Faculdade de Odontologia de Piracicaba PiracicabaSP Brasil Universidade Estadual de Campinas . Faculdade de Odontologia de Piracicaba . Piracicaba , SP , Brasil; II Universidade Estadual de Campinas Faculdade de Enfermagem CampinasSP Brasil Universidade Estadual de Campinas . Faculdade de Enfermagem . Campinas , SP , Brasil

**Keywords:** Diabetes Mellitus, prevention & control, Walking, Intention to Treat Analysis, Randomized Controlled Trial

## Abstract

**OBJECTIVE:**

To evaluate the effect of implementation intentions as an intervention strategy to promote walking in adults with type 2 diabetes mellitus (T2DM).

**METHODS:**

We conducted a controlled and randomized trial, with 12 months of follow-up, involving 65 people with T2DM recruited from primary health care units and allocated them in the control group (CG, n = 32) and intervention group (IG, n = 33). The IG received the implementation intention strategy to promote walking and the CG remained in follow-up for conventional treatment in primary health care. The researchers were blinded by anthropometric measurements and the filling of the instruments.

**RESULTS:**

After twelve months of follow up, the IG presented a statistically significant increase in the leisure time physical activity when compared with the CG (p = 0.0413) and showed a significant decrease in waist circumference (p = 0.0061). No significant difference was observed regarding body mass index and glycated hemoglobin among groups.

**CONCLUSIONS:**

Implementation intention was effective in promoting walking and improving clinical indicators in adults with T2DM.

## INTRODUCTION

There is growing evidence that an epidemic of diabetes mellitus (DM) is occurring worldwide. In 2015, 415 million adults had DM and 318 million had impaired glucose tolerance, placing them at high risk of developing DM in the future ^1^ . About 90-95% of patients with DM manifest type 2 diabetes mellitus (T2DM), which is caused by an interaction between genetic and environmental factors – the latter is related to behaviors, especially sedentary lifestyle, high-fat diets and aging ^2^ .

Pharmacological and non-pharmacological therapeutic possibilities can prevent and control T2DM. The use of non-pharmacological therapy has been proposed as the first option for coping with this disease, improving the quality of life of these individuals and reducing the costs involved in their treatments ^3^ . One of the non-pharmacological treatment options for T2DM is physical activity (PA), which, if performed in a regular and satisfactory manner, promotes greater insulin sensitivity during the first 24 to 72 hours after the exercise session, increasing the glucose in the muscles and reducing blood glucose ^4^ .

According to the American Diabetes Association ^5^ , to prevent complications, the current recommendation of PA for people with DM is 150 minutes of moderate intensity PA spread over at least 3 days/week, with no more than 2 consecutive days without activity. Even though regular PA is essential to prevent complications and improve the quality of life, the frequency of this activity is low in these populations ^6^ .

To better understand the variables involving health behaviors, such as PA, and to propose more effective interventions, researchers have been developing several theoretical sociocognitive models ^7^ . Among them, some motivational models predict that a positive intention is a predictor of behavior ^8^ , such as the Theory of Planned Behavior ^8^ , and Self-efficacy ^9^ . However, although many individuals have a positive intention to act, they fail to transform this intention into behavior; a condition known as a gap in the intention-behavior relationship ^10^ .

Studies suggest this “behavioral failure” occurs because the formulation of a given intention and its execution in the form of a behavior constitute, in fact, two distinct processes ^11^ . From this evidence, a theory ^12^ argues there are two phases for a behavior to be actually executed: 1. the motivational phase, in which the individual constructs a positive intention for the desired behavior and 2. the volitive phase (post-intention). To help people with a positive intention to show a behavior, an intervention strategy called “implementation intention” was developed. In this strategy, the individual formulates plans that specify “when,” “how” and “where” they will show the behavior desired ^12^ . Moreover, the coping planning strategy has been developed simultaneously. The coping planning aims to establish goal development mechanisms to help the individual establish actions to overcome the obstacles experienced in their daily lives. The assumption is that the identification of risk situations is important for the subject to establish effective responses to the problems perceived, i.e., participants realize the barriers and formulate strategies to overcome them. The implementation intention theory has been more effective in promoting health behavior changes than traditional educational strategies ^13^ .

Regarding the effectiveness of interventions based on volitional strategies in the promotion of PA, although some studies have evaluated this aspect ^13 - 15^ none of them evaluated this strategy among patients with T2DM for a period of twelve months of follow-up to test their effect of PA promotion and the improvement of clinical indicators.

The objective of this study was to evaluate the effect of an intervention based on implementation intention as to PA, walking modality and clinical variables among adults with T2DM over a 12-month follow-up period.

## METHODS

This is a randomized two-arm controlled clinical trial, registered in the Brazilian Registry of Clinical Trials (ReBEC): RBR – 6z4t5y. For the method design, the recommendations of the Consolidated Standards of Reporting Trials (CONSORT) were followed regarding trial design, eligibility of the participants and recruitment, study settings, intervention, outcome assessment, sample size, randomization, blinding, participants flow, losses and limitations.

The study was carried out in primary health care units in a small municipality in the state of São Paulo, Brazil. Inclusion criteria were patients diagnosed with T2DM who were older than 18, had medical referral release and positive intention for PA and who were cared for in the five aforementioned units. The diagnosis was based on the information contained in the participant’s health record provided by the attending physician. Those who had a diagnosis of mental disorders (recorded in the medical chart), as well as users of primary care network in clinical conditions that made it impossible for them to perform regular PA were excluded; as well as individuals already practicing it before the intervention. Those who missed one of the face-to-face meetings or who did not perform blood sampling for the glycated hemoglobin (A1C) test were also excluded from the study.

To estimate the sample size, we considered the difference of means and standard deviations of the behavioral variable denominated self-reported behavior, previously validated ^14^ . The sample size was estimated using the paired t-test ^16^ considering two independent samples, based on the minimum expected difference of 1.25, between Control Group (CG) and Intervention Group (IG), on a scale of 0 to 5 points. Thus, a sample of 33 individuals was determined for each group, with a minimum total of 66 participants. We adopted a statistical significance level of 5% and test power of 70%.

To select the sample, an initial survey was carried out to estimate the number of adults with T2DM enrolled in the five health units included in the study. Thus, of the 910 individuals enrolled in the units, 405 individuals met all the inclusion criteria.

After this step, a random allocation list was created, applying a series of random, computer-generated numbers that selected 68 participants among 405 eligible individuals. These were invited by the researcher, via telephone, to participate in the study. At baseline, subjects were randomized into two groups (CG and IG), with 34 participants each.

For the intervention effect assessment, primary outcome was Leisure Time Physical Activity (LTPA), measured through a globally validated questionnaire, as described below. Secondary outcomes were Glycated hemoglobin (A1C), Body Mass Index (BMI), and waist circumference. Outcomes are described in the Measures section.

### Measures

A baseline survey collected the following information of subjects from CG and IG:

1. Leisure Time Physical Activity (LTPA): it was evaluated through the Brazilian version of the Godin-Shephard Leisure-Time Physical Activity Questionnaire (GSLTPAQ), developed in Canada in 1985 and recently adapted to Brazil. ^17^ This is a brief questionnaire to evaluate the frequency and intensity of PA performed in one week. Respondents reported the number of times they would carry on physical activities of vigorous, moderate and light intensity, for at least 15 minutes, usually considering a period of seven days. The frequency indicated was multiplied by an effort coefficient, which is equivalent to the energy expenditure in metabolic equivalents (MET) of the referred activity, generating a score of arbitrary units. Scores were directly proportional to the level of LTPA. After calculating the score, the respondent was categorized as “active,” “moderately active” and “insufficiently active” according to international recommendations, in order to respect the “dose-response relationship,” i.e., the relationship between PA practiced and its health benefits.
2. Sociodemographic and clinical variables were evaluated using a previously validated instrument with questions related to gender, age, income, health institution where the participant was registered and the time of T2DM diagnosis ^18^ .
3. Intention for physical activity: it was measured through a validated instrument ^19^ that assesses the intention of the participant to perform PA. The six-item construct addresses a person’s motivation to carry on a particular behavior, each measured by a Likert scale, with a one to five point response (Example: “I intend to walk for at least 30 minutes three times a week next month”: definitely no [1] – definitely yes [5]). To interpret the scores obtained, the arithmetic mean of the six items that make up the construct was used, so that the higher the average score, the greater the intention of the subject to show that behavior.
4. Anthropometric evaluation: the weight of participants was evaluated using a portable, professional, digital scale. Body mass index (BMI) was estimated based on height and weight measured [BMI (kg/m ^2^ ) = Weight (kg)/Height ^2^ (m)]. Waist circumference (WC) was measured using an inelastic anthropometric tape (in centimeters).
5. Glycated hemoglobin (A1C): it was evaluated by blood quantification using the high-performance liquid chromatography method by a biomedical professional.

### Intervention

The intervention group received the intention implementation strategy. For this, we used two tools called action planning and obstacle coping planning. Action planning is considered the link between the individual’s intention to perform a given behavior and the action itself ^12^ . According to the author of the theory, the execution of a given behavior is activated by certain stimuli that specify “when,” “how,” “where” will be performed. To this end, IG participants were asked to complete a form, with the researcher’s help, in which they should describe a plan of action on when, where and how they intended to walk for at least 30 minutes five times a week (150 min/week), as recommended by the American Diabetes Association ^20^ .

On the other hand, the obstacle-coping plan aimed to establish mechanisms for the development of goals, in order to allow the individual to carry actions aiming to overcome the obstacles experienced in their daily lives. The assumption is that the identification of risk situations for the non-accomplishment of the walk, by the individual, is an important mechanism for the subject to establish effective responses to the problems evidenced ^21^ . IG participants described the barriers to walking and formulated strategies to overcome them.

### Data Gathering Procedures

**Figure 1:**
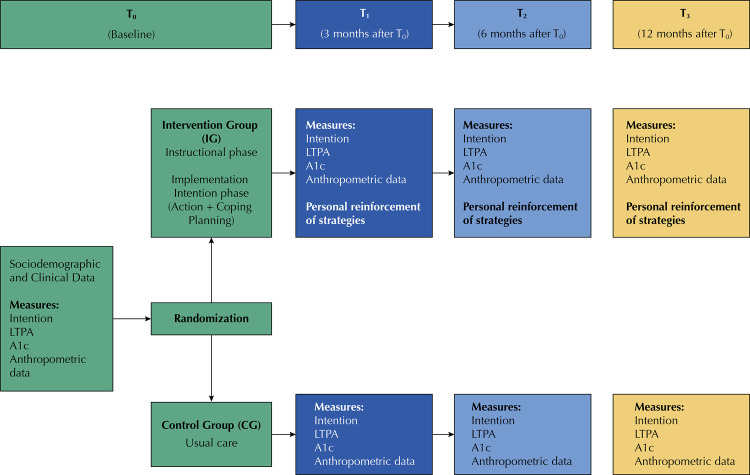
Study design.

Instruments used to gather data were the LTPA, intent for physical activity, anthropometric evaluation and samples, in order to measure A1C in the baseline (T0) and after three (T1), six (T2) and twelve (T3) months of follow-up (see Figure 1 ).

At the first meeting (T0), the subject was invited to participate in the study and had the objectives and procedures of the research explained to them. After accepting to be part of the study, the participants gave their written consent and were randomly sorted in CG and IG. The application of the data collection instruments was performed before any other activity scheduled for this study, in order to avoid the interference of these activities in the measurements in T0 and applied by a trained researcher. In T0, the IG participants received, in a group, the instructional phase of intervention which was intended to inform them on how to effectively perform PA for a diabetic individual, in order to prepare them for subsequent application of the implementation intention strategy. This instructional phase consisted of a teaching-learning strategy through instructional methods of demonstration and execution. Firstly, the method of activating the cognitive domain of the participants was shown through the passive transmission of subjects for 30 minutes: recommendation of 150 minutes of physical activity per week, walking as a means of controlling the disease (30 minutes, 5 times per week) and the importance of stretching before and after these exercises ^20 , 22^ . Then the execution method was performed in order to activate the psychomotor domain of the participants who actively practiced for 30 minutes the main types of stretching and the proper posture of the walk.

After carrying out the instructional phase of the behavior, the participants of the IG proceeded to the implementation phase of the intention by elaborating two plans: action planning and obstacle coping ^22^ previously validated in a previous study on the theme ^14^ .

The CG did not receive any type of intervention but participated in all face-to-face assessments. At the end of the meeting, in T0, all participants of the CG and IG were instructed to take, within seven days after the meeting, a blood sample for the A1C exam, in a laboratory accredited for the research.

In the subsequent meetings (T1, T2 and T3), all participants (CG and IG) were again invited to attend the health units in which they were registered for new anthropometric measures as well as for a new collection of blood for the A1C examination. The behavior regarding the LTPA practice and the intention to practice it were measured again. The IG individuals also received on-site reinforcement of the intention implementation strategy.

All the stages related to the application of the intervention as well as the face-up reinforcement were developed by the principal investigator. The sociodemographic and clinical data, as well as the measurement of the anthropometric measurements and the filling of the instruments, were performed by two other researchers who did not know which individuals belonged to each group (blinding).

### Data Analysis

Initially, analyses were performed to compare IG and CG for sociodemographic and clinical variables. For this comparison, Student’s t-tests were used for age, Wilcoxon-Mann-Whitney test for the time variable of diabetes and Chi-square with Yates correction ^23^ for both gender and income, with a significance level of 5 % (p < 0.05).

For the comparisons between the groups (intergroups) in times and between periods in each group (intragroup), analyses were performed through GEE – Generalized estimating equations ^24^ . In the results that used the GEE models, the estimates obtained from the differences of averages for the continuous quantitative variables, as well as their respective confidence intervals and p-values were presented, and the significance level of 5% was used.

For the intention for physical activity variable, the comparisons between the groups (intergroup) at time T0 and T3 were made using the Mann-Whitney test. ^23^ As for the comparisons between periods, in each group (intragroup) the Wilcoxon Rank-Sum Test Soma test was applied ^23^ . In the case of this variable, four tests (two intragroup and two intergroup) were performed and, for this reason, the Bonferroni correction was applied at a significance level of 1.25% (p < 0.0125).

### Ethical Aspects

The research was approved by the local Research Ethics Committee (CAAE: 35399914.2.0000.5418) and was conducted in accordance with the principles of the Declaration of Helsinki.

## RESULTS

**Figure 2:**
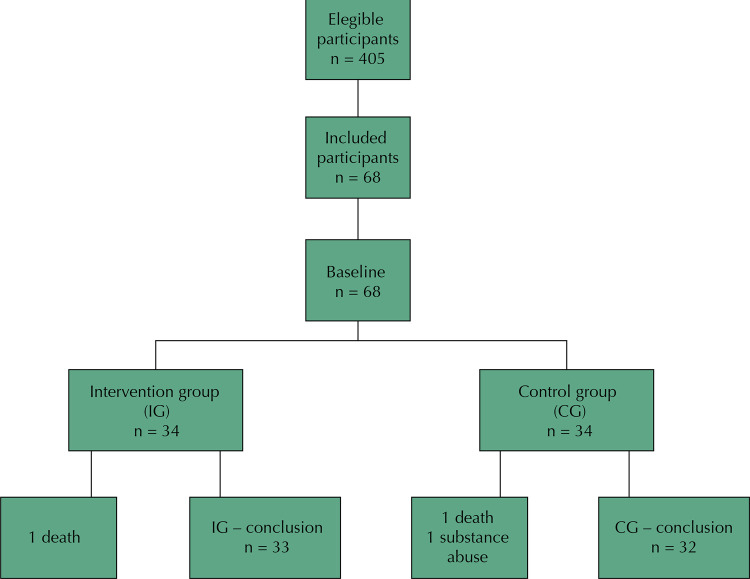
Study flowchart and sample constitution. Dois Córregos, SP, Brazil, 2016.

The study flow chart is presented below. Over the twelve months of intervention, there were three patients lost to follow-up (CG – two losses due to death and use of drugs/IG – one loss due to death – see Figure 2 ).

Table 1 presents the description of the sociodemographic and clinical variables of the study participants in CG and IG. We found that the number of women who participated in the study was higher in both study groups, and they have been living with the disease for over seven years. We verified that the mean age of male participants was 62.4 (SD: 10.77). No significant difference was found between groups for the sociodemographic and clinical variables studied.

**Table 1:** Description of the groups regarding sociodemographic and clinical variables at baseline, *Dois Córregos* , SP, Brazil, 2016.

Variables	Control group (n = 32)	Intervention group (n = 33)	p
Age (mean, (SD))	63.25 (10.33)	60.21 (10.83)	0.2518
Time since diabetes diagnosis (years – means (SD)	7.78 (5.62)	9.36 (6.47)	0.4010
Gender, male (%)	40.62	24.24	0.2515
Gender, female (%)	59.38	75.76	
Family income (≤ BMW, %)	37.50	27.27	0.5377
Family income (> BMW, %)	62.50	72.73	

SD: standard deviation; BMW: Brazilian minimum wage ≈ US$ 251.00

The intragroup comparison analysis showed a statistically significant increase in LTPA levels between T0 and T2 (p = 0.0147) and T0 and T3 (p = 0.0413). Intergroup analyses, however, showed a significant difference in T2 time in favor of the intervention group (p = 0.0407) ( Table 2 ).

**Table 2:** Comparison of the behavioral variable between follow-ups in the intervention and control groups, *Dois Córregos* , SP, Brazil, 2016.

		Standard deviation (95%)			p ^a^
		Mean difference	Inferior limit	Superior limit	
GSLTPAQ ^b^	T1 - T0 (CG)	3.34	-4.18	10.87	0.3836
	T2 - T0 (CG)	-0.41	-8.62	7.81	0.9228
	T3 - T0 (CG)	1.75	-5.18	8.68	0.6204
	CG - IG (T0)	-0.69	-10.18	8.80	0.8868
	CG - IG (T1)	-0.92	-7.10	5.26	0.7702
	CG - IG (T2)	**-7.49**	**-14.66**	**-0.32**	**0.0407**
	CG - IG (T3)	-5.09	-12.50	2.32	0.1781

^a^ p-value obtained through GEE models - Generalized estimating equations. ^b^ Godin-Shephard Leisure-Time Physical Activity Questionnaire. Note: In these cases, the null hypothesis was rejected when the p-value was less than 0.05.

Regarding the clinical variables, the intragroup comparison analyses showed that the people submitted to the intervention presented a statistically significant reduction of WC between the times T0 and T1 (p = 0.0141), T0 and T2 (p = 0. 0151) and T0 and T3 (p = 0.0061) ( Table 3 ). However, for the variables BMI and A1C, although they presented reduction over all times in the IG, this reduction was not statistically significant.

**Table 3:** Comparisons of the clinical variables between IG and CG at different times, *Dois Córregos* , SP, Brazil, 2016.

Variable	Comparison	Mean difference	Standard deviation (95%)		p ^*^
			Superior limit	Inferior limit	
Body mass index (BMI)	T1 - T0 (CG)	0.06	-0.23	0.36	0.6870
	T2 - T0 (CG)	0.34	-0.05	0.72	0.0849
	T3 - T0 (CG)	0.11	-0.34	0.57	0.6227
	T1 - T0 (IG)	-0.26	-0.63	0.12	0.1777
	T2 - T0 (IG)	-0.29	-0.68	0.10	0.1469
	T3 - T0 (IG)	-0.21	-0.57	0.16	0.2604
	CG - IG (T0)	-0.17	-3.10	2.76	0.9112
	CG - IG (T1)	0.15	-2.75	3.06	0.9188
	CG - IG (T2)	0.46	-2.49	3.42	0.7592
	CG - IG (T3)	0.16	-2.89	3.20	0.9197
Waist circumference (WC)	T1 - T0 (CG)	-0.50	-2.78	1.78	0.6676
	T2 - T0 (CG)	1.34	-0.90	3.59	0.2414
	T3 - T0 (CG)	-0.59	-2.84	1.65	0.6040
	T1 - T0 (IG)	**-1.64**	**-2.94**	**-0.33**	**0.0141**
	T2 - T0 (IG)	**-1.61**	**-2.90**	**-0.31**	**0.0151**
	T3 - T0 (IG)	**-2.03**	**-3.48**	**-0.58**	**0.0061**
	CG - IG (T0)	-2.02	-8.16	4.11	0.5178
	CG - IG (T1)	-0.89	-6.72	4.94	0.7656
	CG - IG (T2)	0.93	-5.02	6.87	0.7602
	CG - IG (T3)	-0.59	-6.73	5.56	0.8514
Glycated hemoglobin (A1C)	T1 - T0 (CG)	0.19	-0.15	0.53	0.2649
	T2 - T0 (CG)	0.49	-0.13	1.12	0.1213
	T3 - T0 (CG)	0.53	-0.13	1.18	0.1158
	T1 - T0 (IG)	-0.02	-0.41	0.37	0.9219
	T2 - T0 (IG)	-0.01	-0.47	0.45	0.9562
	T3 - T0 (IG)	-0.27	-0.87	0.33	0.3800
	CG - IG (T0)	-0.30	-1.25	0.66	0.5445
	CG - IG (T1)	-0.08	-1.13	0.96	0.8766
	CG - IG (T2)	0.21	-0.89	1.31	0.7077
	CG - IG (T3)	0.50	-0.46	1.46	0.3096

* p-value obtained through GEE models - Generalized estimating equations. Note: In these cases, the null hypothesis was rejected when the p-value was less than 0.05.

In the intra- and intergroup analyses for the intention variable, between T0 (baseline) and T3 (12 months), no significant difference was observed in CG and IG (p > 0.0125), showing homogeneity of this variable in both groups. It was also found that the mean of this construct was above half (2.5) at T0 and T3 times between CG individuals (T0 = 3.76 and T3 = 3.68) and IG (T0 = 4.14 and T3 = 4.3), evidencing that all participants had a positive intention about the practice of physical behavior from the baseline ( Table 4 ).

**Table 4:** Comparisons of the intention for physical activity variable between the times in the intervention and control groups, *Dois Córregos* , SP, Brazil, 2016.

Variable	Group	Time	N	Mean	Standard Deviation	Median	Comparison	p
Intention	CG	T0	32	3.76	1.18	4	Group CG-IG (T0) ^a^	0.2483
		T3	32	3.68	1.28	4	Group CG-IG (T3) ^a^	0.0323
	IG	T0	33	4.14	0.93	4	T0-T3 (Group CG) ^b^	0.8749
		T3	33	4.3	1.14	5	T0-T3 (Group IG) ^b^	0.3397

^a^ p-value obtained by means of the Mann-Whitney test. ^b^ p-value obtained through the Wilcoxon Signal-Sum test. Note: In these cases, the null hypothesis was rejected when the p-value was lower than 0.0125.

## DISCUSSION

This study evaluated the impact of a behavioral intervention based on the implementation intention on the practice of PA in adults with T2DM treated in primary health care. The results showed that the participants submitted to the intervention had a statistically significant increase in the LTPA frequency after six and twelve months of intervention, compared with CG. There was probably no significant increase in this variable in IG in the first three months as those were the coldest three months of the year in Brazil, and cold weather may be an obstacle in the execution of outdoor PA, such as walking ^25^ .

The increase in PA behavior after six and twelve months, in turn, can be associated with the practice of walking, as the instrument used to measure such behavior generally adds all types of LTPA and this was the physical modality recommended to the participants. Significant increase in frequency for such behavior probably occurred voluntarily, corroborating previous studies that use this type of intervention and also observed an increase in the practice of PA ^14 , 15 , 26^ .

Regarding the follow-up period, to date, no other studies have used the intention implementation strategy to promote PA practice for such a long time (12 months). The period in question points to the probable acquisition of automaticity characteristics of said behavior, becoming a healthy habit by repetition in appropriate contexts ^27^ . This automaticity is highly desirable as it allows the participant to maintain adherence to regular practice of PA as one of the pillars of T2DM treatment, even if there was no intervener to guide it.

However, even with the increase in adherence to the physical behavior in the IG, no significant reductions in BMI were observed during the 12-month follow-up period. This can be explained by the fact that we have directed efforts to PA and not to other behaviors that could also contribute to weight loss. For example, a study that applied an intervention that included PA along with nutritional guidelines had a statistically significant reduction of BMI ^28^ .

Regarding waist circumference (WC), the increase in LTPA behavior in the IG was accompanied by a statistically significant reduction in this indicator, corroborating with previous findings in which a diabetic population also significantly reduced their WC after a period of two months of this kind of intervention ^26^ . This finding in the IG is of fundamental importance for public health, since abdominal fat is closely related to the presence of insulin resistance, and these two factors together lead to an increase in mortality in this population, mainly of cardiovascular origin ^29^ . It is noteworthy that reductions in abdominal fat, even without BMI reduction, can have a positive impact on the cardiometabolic profile, making WC one of the most reliable anthropometric measures in the therapeutic control of diabetic patients ^30^ .

Although the A1C variable did not show a significant reduction between the follow-ups in the IG, there was a mean decrease in their values at all times in the IG; this was not observed in CG, as it showed a worsening in relation to this variable. This was probably due to the absence of an intervention in the caloric intake of diabetics allied with the intervention for the practice of walking.

Due to the fact that it is a study that seeks the control of diabetes, interventions on behavior and lifestyle are necessary, especially with the inclusion of regular practice of PA and a reorganization of eating habits; this study did, however, only intervene in promoting the practice of the former. It is also worth noting the use of self-report instruments that, due to their subjective nature, present considerable limitations (such as memory bias), making it necessary to combine them with objective instruments such as accelerometers, pedometers and motion sensors to accurately measure PA levels. Another limitation that should be scored is the reduced number of participants, possibly harming the external validity and applicability of the study.

However, despite their limitations, the results obtained provide an important theoretical basis for directing multiprofessional interventions related to the adoption of an active lifestyle by individuals with T2DM, an important gap in nursing care and other related professions.

Regarding the strengths of the study and its application in the clinical setting, we highlight that there are few theoretical-based intervention studies that aim to promote LTPA to people with diabetes mellitus. Therefore, considering that the sedentarism is a worrying behavior and may have implications on metabolic control, quality of life and emotional aspects of diabetic patients, this study proposes an innovation, since it provides to nurses, physicians and other health professionals a method of intervention that is brief, low cost, effective and that can be easily offered to patients undergoing primary care by combining behavioral strategies.

## CONCLUSION

The Implementation Intention Theory was effective to promote walking and positive changes on the clinical measurements among people with T2DM over a 12-month follow-up period.
